# Associations between obesity and mental distress in late midlife: results from a large Danish community sample

**DOI:** 10.1186/s40608-016-0137-x

**Published:** 2016-12-12

**Authors:** Cathrine Lawaetz Wimmelmann, Rikke Lund, Ulla Christensen, Merete Osler, Erik Lykke Mortensen

**Affiliations:** 1 Department of Public Health, Medical Psychology Unit, University of Copenhagen, Center for Healthy Aging, Østerfarimagsgade 5A, Building 5, 1. Floor, 1353 Copenhagen K, Denmark; 2 Department of Public Health, University of Copenhagen, Copenhagen, Denmark; 3 Center for Healthy Aging, Faculty of Health and Medical Sciences, University of Copenhagen, Copenhagen, Denmark; 4 Research Center for Prevention and Health, Glostrup Hospital, Glostrup, Denmark; 5 Danish Aging Research Center, Universities of Aarhus, Southern Denmark, Copenhagen, Denmark

**Keywords:** Obesity, BMI, Psychology, Mental distress, SCL-90, Socio-demographic factors, Danish population

## Abstract

**Background:**

To examine associations of Body mass Index (BMI) and mental distress in late midlife in a large Danish community sample and to investigate the effect of socio-demographic factors.

**Methods:**

The study sample comprised 3613 Danish men and 1673 women aged 49–63 years from the Copenhagen Ageing and Midlife Biobank (CAMB) with complete information on measured BMI, severity of mental symptoms assessed by the Symptom Check-List’ (SCL-90), and socio-demographic factors including sex, age, occupational social class, and educational duration. Linear and logistic regression were used to evaluate associations between BMI category and SCL-90.

**Results:**

Unadjusted SCL-90 subscale scores differed significantly across BMI categories (*p* < 0.001) among both men and women with more mental distress in the underweight, obese and severely obese BMI categories except for the anxiety scale which was not associated with BMI category in women. In the adjusted analyses, all symptom scales remained significantly associated with BMI among men after adjusting for socio-demographic factors while only associations with somatization and depression scales remained significant for women.. When SCL-90 case status was applied as an outcome, significant unadjusted associations with BMI category were observed for somatization (*p* < 0.001), depression (*p* = 0.026) and the General Severity Index (*p* = 0.002) among men and somatization (*p* = 0.002) among women. Furthermore, somatization case-status was significantly predicted by BMI category (*p* < 0.001) in men after adjusting for socio-demographic factors.

**Conclusion:**

Results indicate more mental distress among underweight, obese and severely obese men and women after adjusting for socio-demographic factors. Furthermore, obese men have higher risk of reporting clinically relevant symptoms of somatization independently of socio-demographic factors.

## Background

Obesity and mental health disorders constitute two major public health concerns. A growing body of evidence has suggested that obesity is associated with elevated levels of mental distress [1–3] and that obese individuals have an increased risk of developing psychiatric disorders [1]. However, in general findings are inconsistent and especially previous research has provided conflicting results possibly explained by methodological differences across studies [4]. Though the majorities of more recent studies [1, 3, 5] have shown that obesity is strongly associated with mental distress and psychiatric disorders, some studies have found no relation [6] or inverse relationships in men [7, 8]. Also, longitudinal studies suggest that obesity may be both a cause and a consequence of mental distress [9–11]. It is very likely that the relation between mental distress and obesity is reciprocally creating a viscous circle: factors such as impaired physical function and obesity-related stigmatization may result in elevated mental distress in obese individuals while inappropriate eating behaviours including emotional eating and comfort eating may lead to obesity in individuals with poor mental health. In general, the inconsistent results found in the literature suggest that the relation between mental distress and obesity is complex and remains unclear.

Socio-demographic factors including sex, age and educational status have been proposed as potential moderators of the obesity-mental health relation [5, 12, 13]. Few studies have investigated the influence of socio-demographic factors on the association between obesity and mental distress in the general adult population. The majority of these studies [5, 13–15] have found associations between obesity and mental symptoms to be stronger in women than in men. Simon et al. [1] reported that sex did not moderate the relation between obesity and mental distress, while Zhao et al. [3] reported that obesity was associated with psychiatric disorders among underweight and severely obese men and overweight and obese women. Assari et al. [13] found that among 3.648 black and white adults both sex, age and race moderated the association between BMI and psychosocial factors. More specifically, high BMI was associated with poor mental quality of life among older white women, and older black men and women while high BMI was associated with poor physical quality of life in all demographic groups.

Other studies [1, 5, 16, 17] have found evidence that association of obesity with mental distress may be stronger in individuals from higher social classes, white populations and in those with higher educational attainment possibly reflecting stronger perceived obesity-related stigmatization in socio-demographic groups with low obesity prevalence. However, the effect of age and educational level seems to vary depending on the nature of the symptoms [5].

In the literature, a range of standardized questionnaires has been used to assess mental distress. Symptom Checklist 90 (SCL-90) [18] is a well-validated symptom inventory assessing different aspects of mental distress and has been used extensively in clinical research. However, to date no large community based studies have reported SCL-90 scores across BMI categories. Publication of such information is of major importance and highly needed within several branches of the obesity research. That is, in addition to providing knowledge about mental distress across BMI groups, community based BMI specific SCL-90 scores would also serve comparative purposes for evaluating obese subpopulations such as bariatric patients.

The aims of the present study therefore were to examine associations of BMI and mental distress assessed by the SCL-90 in late midlife men and women and to investigate whether socio-demographic factors confound or modify the relation between BMI and mental distress. And finally, to describe mental distress across BMI groups in a community based population.

## Methods

### Study sample

The study aims were conducted using data from the Copenhagen Ageing and Midlife Biobank (CAMB), which is based on follow-ups of members from three longitudinal cohorts [19], The Metropolit Study [20], The Copenhagen Perinatal Cohort [21] and The Danish Longitudinal Study on Work Unemployment and Health [22]. CAMB as a database combining these three cohorts was approved by the regional research ethical committee (No: H-A-2008–126). Initially, more than 5.500 participants were assessed at the late midlife follow-ups corresponding a response rate of 30%. Complete information about measured BMI and socio-demographic factors including sex, age, years of education and social class was available for 5417 participants. Data on mental distress were missing for 131 of these participants. Thus, the final study sample comprised 5286 participants of which 3613 are men and 1673 are women.

### Body mass index

Both height and weight were measured at the clinical examination [23]. Body mass index (BMI) of the participants was calculated as weight (kg)/(height (m))^2^ and BMI was recoded into a categorical variable with five BMI groups based on the World Health Organization (WHO) standards. The five BMI groups are ‘Underweight’ (BMI < 18.5 kg/m^2^), ‘Normal weight’ (BMI 18.5–24.9 kg/m^2^), ‘Overweight’ (25–29.9 kg/m^2^), ‘Obese’ (30–39.9 kg/m^2^), ‘Severely obese’ (BMI > 40 kg/m^2^).

### Outcome measures

#### Mental distress

*The* ‘*Symptom Check*-*List*’ (*SCL*-*90*) [18] consists of 90 items and is used to assess the severity of a variety of a wide range of mental symptoms. In the CAMB data collection, a shorter version comprising 35 items scored in a 0–4 Likert format was used to obtain information on the three subscales somatization, depression, and anxiety symptoms with higher scores indicating more mental distress. Also, a fourth scale - the General Severity Index (GSI) - was calculated as the mean item score across the three subscales. Both the subscale scores and the Danish cut-off values for SCL-90 case status calculated by Olsen et al. [24] were used in the present study. Cut-off values for the GSI (based on the three included subscales) corresponding to a T-score of 63 were 1.29 for women and 1.01 for men. The subscales were used as continuous variables in linear regression analyses, and in the dichotomous version (case/non case) in logistic regression analyses.

### Socio-demographic variables

#### Sex

By design the three cohorts included in the CAMB sample have different sex distributions. The Metropolit study cohort consists of men only, while the Copenhagen perinatalcohort and the Danish Longitudinal Study on Work Unemployment and Health cohort include both men and women. The total sample of the present study consists of 3613 (68.4%) men and 1673 women (31.6%).

#### Age

The age range of the total sample was 49–63. By design the three cohorts included in the CAMB sample differ with regard to age and two subsamples were derived. One subsample aged 49–53 years (from the Copenhagen perinatal cohort and the Danish Longitudinal Study on Work Unemployment and Health cohort) and a second subsample aged 56–63 years (from the Metropolit study cohort and the Danish Longitudinal Study on Work Unemployment and Health cohort). Thus, age was analysed as a binary variable indicating these two subsamples (see Mortensen et al. [25]).

### Occupational social class

The social class of the participants was classified into six categories based on their occupational level. The six social classes correspond the standards of the Danish occupational social class classification [26] with social class I-V comprising occupationally active individuals in positions requiring high educational attainment (class 1) to individuals in unskilled work (class V). Social class VI represents individuals on transfer income including sickness benefits and disability pension. Finally, a seventh social class category labelled “Other” included individuals with insufficient occupational information, students and housewives.

### Education in years

‘Education in years’ provides information about the duration of the participants’ education on an 8–17 years scale. This quantitative variable was derived from two categorical variables: School education and Vocational training. Three categories of school education (low, medium, high) were recoded to 8–12 years and three vocational training categories to 0–5 years, combined resulting in the applied scale of educational duration. For a detailed description see Mortensen et al. [27].

### Data analysis

Descriptive statistics were used to investigate participant characteristics and differences across BMI groups were examined using chi-square tests or analyses of variance (ANOVA) depending on the nature of the dependent variable. Associations of BMI category with SCL-90 were investigated in both linear and logistic regression models. Preliminary analyses showed a significant interaction between sex and BMI category with respect to the somatization score (*p* = 0.003) and a marginally significant interaction between sex and BMI category for somatization case status (*p* = 0.054). Therefore, associations between BMI category and the continuous scores of the SCL-90 subscales were analysed separately for men and women using ANOVA in unadjusted models and ANCOVA in models adjusting for age, and occupational social class as categorical covariates and duration of education as a continuous covariate.

Furthermore, to investigate the prevalence of potentially clinically relevant SCL-90 scores across BMI categories, cut-off values for SCL-90 case status were applied and case status used as outcome in logistic regression analyses. Again, unadjusted models and adjusted models including age, occupational class, and years of education were analysed separately for men and women.

Finally, to investigate whether any of the socio-demographic covariates moderated associations between BMI category and SCL-90 score or SCL-90 case status, interaction effects of each covariate with BMI category were included as product terms separately in both the linear and the logistic regression models.

## Results

Table 1 presents participant characteristics including, sex, age, occupational social class, and years of education for the total sample and for each BMI category. The five BMI groups differed on all characteristics. The underweight group included a significantly larger proportion of women and was also younger than the groups with higher BMI. With respect to occupational social class the obese and severely obese groups had the largest proportion of participants in the lowest social class V and on transfer income. Finally, the two obese groups reported shorter education than the rest of the BMI groups.

**Table 1:** Socio-demographic characteristics of the total Copenhagen Aging and Midlife Biobank (CAMB) sample and across body mass index categories

	Total	Under weight	Normal weight	Overweight	Obese	Severely obese	*P* value^1^
N	5286	55	2243	2198	746	44	
Sex N (%)							
- Men	3613 (68)	16 (29)	1356 (61)	1676 (76)	541 (73)	24 (54)	<0.001
- women	1673 (32)	39 (71)	887 (39)	522 (24)	205 (27)	20 (46)	
Age N (%)							
- Age 49–53	2262 (43)	34 (62)	1067 (48)	826 (38)	311 (42)	24 (54)	<0.001
- Age 56–63	3024 (57)	21 (38)	1176 (52)	1372 (62)	435 (58)	20 (46)	
Social class N (%)							
- I	826 (16)	10 (18)	407 (18)	330 (15)	76 (10)	3 (7)	<0.001
- II	1376 (26)	13 (24)	597 (27)	576 (26)	182 (24)	8 (18)	
- III	1231 (23)	6 (11)	506 (23)	541 (25)	168 (22)	10 (23)	
- IV	870 (16)	9 (16)	361 (16)	362 (16)	131 (18)	7 (16)	
- V	447 (8)	6 (11)	164 (7)	183 (8)	89 (12)	5 (11)	
- Transfer income	501 (10)	11 (20)	189 (8)	197 (9)	94 (13)	10 (23)	
- Other	35 (1)	0 (0)	19 (1)	9 (1)	6 (1)	1 (2)	
Education in years M (SD)	13.2 (2.4)	13.4 (2.7)	3.6 (2.4)	13.0 (2.4)	12.4 (2.3)	12.1 (2.2)	<0.001

Note: ^1^ *P* value for chi-square tests (categorical variables) or F tests (continuous variables) of no significant difference between the BMI categories

Mean (*M*), Standard deviation (*SD*)

Results from the linear analyses of associations between BMI category and mental distress are shown in Table 2. SCL-90 subscale means for the total CAMB sample were slightly lower than Danish norms [24] (data not shown). For both men and women significant group differences were observed across BMI categories on all four SCL-90 subscales with a U – shaped relation between BMI category and SCL-90 score on all subscales except anxiety that did not differ significantly across BMI groups for women. Thus, together with the underweight men and women,, the obese groups displayed higher scores on somatization, depression and the GSI than the normal weight BMI group. Overweight men displayed the lowest symptom load on all subscales whereas the normal weight group reorted the lowest level of mental distress among women. These differences remained highly significant in the adjusted models with age, occupational social class and duration of education as covariates. For men, all covariates except age group significantly predicted the somatization score (*P* < 0.001) while only occupational social class contributed siginifanctly to the depression, the anxiety and the GSI scales (*p* < 0.001). For women, occupational social class was significantly associated with all four SCL-90 subscales (*p* < 0.001), age group significantly predicted the somatization, the depression and the GSI scales (*p* < 0.05), whereas duration of education was associated with somatization (*p* < 0.05).

**Table 2:** Analyses of variance of associations between BMI category and mental distress assessed with the four SCL-90 subscales

Men	Total	Underweight		Normal weight		Overweight		Obese		Severely obese		F-statistic		*P*-value	
	Unadj. M (SD)	Unadj. M (SD)	Adj. M	Unadj. M (SD)	Adj. M	Unadj. M (SD)	Adj. M	Unadj. M (SD)	Adj. M	Unadj. M (SD)	Adj. M	Unadj. M (SD)	Adj. M	Unadj.	Adj.
Somatization	0.34 (0.4)	0.45 (0.5)	0.46	0.32 (0.4)	0.38	0.31 (0.3)	0.35	0.45 (0.5)	0.47	0.51 (0.4)	0.50	16.68	10.72	<0.001	<0.001
Depression	0.39 (0.5)	0.53 (0.4)	0.51	0.38 (0.5)	0.41	0.37 (0.5)	0.40	0.49 (0.5)	0.49	0.53 (0.6)	0.51	8.45	5.29	<0.001	<0.001
Anxiety	0.35 (0.4)	0.49 (0.5)	0.45	0.35 (0.4)	0.35	0.33 (0.4)	0.32	0.38 (0.4)	0.36	0.35 (0.4)	0.24	4.43	3.46	<0.05	<0.05
GSI	0.36 (0.4)	0.49 (0.4)	0.48	0.35 (0.4)	0.38	0.34 (0.4)	0.36	0.44 (0.4)	0.45	0.45 (0.4)	0.43	10.66	6.81	<0.001	<0.001
Women															
Somatization	0.47 (0.5)	0.46 (0.5)	0.51	0.41 (0.4)	0.48	0.51 (0.5)	0.55	0.58 (0.6)	0.61	0.61 (0.6)	0.53	7.59	4.86	<0.001	<0.001
Depression	0.52 (0.5)	0.61 (0.6)	0.72	0.47 (0.5)	0.61	0.52 (0.5)	0.65	0.59 (0.5)	0.70	0.61 (0.5)	0.61	3.95	5.29	<0.05	<0.05
Anxiety	0.40 (0.5)	0.45 (0.5)	0.55	0.38 (0.4)	0.50	0.39 (0.4)	0.49	0.43 (0.5)	0.52	0.40 (0.4)	0.41	1.12	0.84	NS	NS
GSI	0.46 (0.4)	0.51 (0.5)	0.60	0.42 (0.4)	0.53	0.48 (0.4)	0.57	0.54 (0.5)	0.62	0.55 (0.4)	0.52	4.14	2.20	<0.05	NS

Unadjusted mean (M) and standard deviation (SD) and adjusted mean with age, occupational social class and years of education included as covariates

Note: All independent factors except duration of education were analyzed as categorical variables. General Severity Index (GSI)

For the total CAMB sample, the prevalence of SCL-90 case status was 7.0% for the somatization subscale, 5.6% for depression, 7.0% for anxiety and 5.8% for the GSI. Results from the logistic regression analyses are presented in Table 3 showing prevalence of SCL-90 case status and unadjusted and adjusted odds ratios across BMI categories. For both men and women, the highest prevalence of SCL-90 cases was observed among either the two obese groups or the underweight group on all subscales. Among men, the unadjusted logistic regression analyses showed significant associations between BMI category and case status for all SCL-90 subscales except anxiety which was only borderline significant (*p* = 0.060). However for women, associations of BMI category with clinical relevant symptoms were significant for somatization only. Unadjusted odds ratios showed that for somatization, depression and the GSI subscales men and women in either the obese or severely obese group had the highest risk of case status, and underweight men had the highest risk of case status on the anxiety subscale. In contrast, the overweight men did not have significantly higher risk of case status on any subscale (unadjusted odds ratio for the overweight men ranged from 0.8 (0.7; 1.1) to 1.0 (0.8; 1.4)). The association between BMI category and SCL-90 case status remained significant for somatization only in men after adjusting for age, occupational social class and duration of education and none of the subscales were significantly associated with BMI category in women in the adjusted analyses. Furthermore, occupational social class was the only predictor that was significantly associated with all SCL-90 subscales after including socio-demographic covariates. More specifically, the transfer income group had significantly higher risk of mental distress among both men and women (adjusted odds ration ranged from 5.6 (3.3; 9.6) to 12.4 (6.3; 24.9) *p* < 0.001 for men and 6.0 (2.2;16.2) to 46.3 (6.0;358.0) for women when social class I was used reference).

**Table 3:** Logistic regression of associations between BMI category and mental distress case status (assessed by the four SCL-90 subscales)

	Total sample %case	Underweight			Normal weight			Overweight			Obese			Severely obese			*P*-value^1^	
Men		%case	OR (CI)	Adj. OR (CI)	%case	OR (CI)	Adj. OR (CI)	%case	OR (CI)	Adj. OR (CI)	%case	OR (CI)	Adj. OR (CI)	%case	OR (CI)	Adj. OR (CI)	OR	Adj. OR
Somatization	7.3	11.3	1.7 (0.7;3.8)	1.2 (0.5;2.9)	7.0	1.0 (ref)	1.0 (ref)	5.4	0.8 (0.6;1.0)	0.7 (0.5;0.9)	13.5	2.1 (1.5;2.9)	1.7**(1.2;2.4)	16.7	2.7 (0.9;8.0)	1.8 (0.6;5.9)	0.000	0.000
Depression	5.8	4.	0.9 (0.3;3.0)	0.7 (0.2;2.2)	5.2	1.0 (ref)	1.0 (ref)	5.3	1.0 (0.7; 1.4)	1.0 (0.7;1.4)	8.9	1.8 (1.2;2.6)	1.5*(1.0;2.3)	8.3	1.7 (0.4;7.2)	1.3 (0.3;6.2)	0.026	0.163
Anxiety	7.1	12.9	1.8 (0.8;3.9)	1.4 (0.6;3.2)	7.5	1.0 (ref)	1.0 (ref)	6.1	0.8 (0.6;1.1)	0.8 (0.6;1.0)	9.1	1.2 (0.9;1.8)	1.1 (0.7;1.5)	0.0	---	---	0.060	0.218
GSI	6.0	8.1	1.5 (0.6;3.8)	1.0 (0.4;2.7)	5.6	1.0 (ref)	1.0 (ref)	5.1	0.9 (0.7;1.3)	0.9 (0.6;1.2)	9.8	1.8 (1.3;2.7)	1.5* (1.0;2.2)	4.2	0.7 (0.1;5.5)	0.5 (0.1;3.6)	0.002	0.061
Women																		
Somatization	6.4	7.5	2.0 (0.9;4.0)	1.6 (0.7;3.5)	4.0	1.0 (ref)	1.0 (ref)	7.6	2.0 (1.2;3.2)	1.7* (1.0;2.9)	10.7	2.9 (1.6;5.1)	2.2**(1.2;4.2)	15.0	4.3 (1.2;15.3)	1.5 (0.4;6.5)	0.002	0.101
Depression	5.3	7.5	1.8 (0.9;3.6)	1.3 (0.6;2.9)	4.4	1.0 (ref)	1.0 (ref)	5.3	1.2 (0.7; 2.0)	1.1 (0.6;1.9)	6.3	1.5 (0.8;2.9)	1.1 (0.6;2.3)	10.0	2.4 (0.5;10.9)	1.0 (0.2;5.1)	0.399	0.948
Anxiety	6.6	6.8	1.0 (0.5;2.1)	0.8 (0.4;1.7)	6.7	1.0 (ref)	1.0 (ref)	6.1	0.9 (0.6;1.4)	0.8 (0.5;1.3)	7.8	1.2 (0.7;2.1)	0.9 (0.5;1.7)	5.0	0.7 (0.1;5.6)	0.3 (0.1;2.4)	0.940	0.693
GSI	5.4	6.8	1.8 (0.9;3.7)	1.4 (0.6;3.0)	4.0	1.0 (ref)	1.0 (ref)	6.1	1.6 (0.9;2.6)	1.4 (0.8;2.4)	7.3	1.9 (1.0;3.6)	1.5 (0.7;2.9)	10.0	2.7 (0.6;12.1)	1.0 (0.2;5.2)	0.176	0.706

Unadjusted odds ratio and adjusted odds ratio with age, occupational social class, and duration of education as covariates

Note: All independent factors except duration of education were analyzed as categorical variables. Odds ratio (OR); Confidence interval (CI)

^1^ *P* value of associations between BMI category and SCL-90 subscale case status

*Difference from reference category remained significant at *p* < 0.05 in adjusted model

**Difference from reference category remained significant at *p* < 0.001 in adjusted model

Few moderation effects were observed for the socio-demographic covariates. In the linear regression models, the interaction between occupational social class and BMI category was significant for somatization (*p* = 0.030), depression (*p* = 0.047) and GSI (*p* = 0.050) in men with a stronger association between BMI group and SCL score among men in social class I and on transfer income. For women, the interaction between occupational social class and BMI category was significant for somatization (*p* = 0.009), anxiety (*p* = 0.015) and GSI (*p* = 0.048) with a stronger association between BMI group and SCL score for women in social class VI and on transfer income. There were no moderation effects of the socio-demographic covariates in any of the logistic models.

## Discussion

To our knowledge this is the first large Danish community based study to investigate the association between BMI category and mental distress assessed with the SCL-90 somatization, depression, anxiety, and GSI subscales.

We found significant differences on all four SCL-90 subscales across BMI categories. Except for anxiety, both obese and severely obese individuals reported more mental symptoms than the normal weight group and these differences remained significant after adjusting for socio-demographic factors. For women the normal weight group had the lowest symptom load on all SCL-90 scales whereas overweight men did not differ significantly from their normal weight counterparts on any of the subscales. This emphasizes the importance of distinguishing between overweight and obese individuals as they mayrepresent two distinct groups with regard to mental distress. Also, in the present study underweight men and women displayed elevated levels of mental distress comparable to that of obese and severely obese individuals. These results (Table 2) are in line with prior research showing a U-shaped relation between BMI and common mental disorders [3, 28–30] suggesting that both underweight and obese individuals experience more mental distress compared with their normal weight and overweight counterparts. Prior research has suggested several reasons for the elevated mental distress observed in obese individuals including stigmatization, social undesirability, and dissatisfaction with appearance. [31, 32] Also, medical comorbidities and impaired physical function are prevalent in both underweight and obese individuals and have consistently been associated with poor mental health [33–37]. Both underweight and obesity may have pathological causes including eating disorders and medical disease. Thus, the elevated level of mental distress in underweight and obese men and women observed in the present study may reflect underlying pathology or physical impairments. Furthermore, the consistent associations between the extreme BMI categories and the somatization subscale observed in the present study may partly reflect such physical impairments as this subscale assess the tendency to experience and communicate unexplained medical symptoms.

Until now community based SCL-90 scores across BMI categories have been lacking complicating the interpretation of SCL-90 scores within specific obese subpopulations such as bariatric patients. The sex-specific SCL-90 subscale scores presented in Table 2 provide such community based BMI specific SCL-90 scores useful in future evaluation of obese subpopulations.

When case status on the four SCL-90 subscales was applied as outcome, BMI category significantly predicted somatization, depression and GSI in men and somatization only among women. The obese and severely obese groups were up to 2.7 (for men) and 4.3 (for women) times more likely of displaying clinical significant symptoms (case status) than their normal weight counterparts. However, socio-demographic factors, especially occupational social class, explained most associations between BMI category and SCL-90 case status as reflected by non-significant associations in adjusted models that included occupational social class as covariate. In men BMI category remained a significant predictor of somatization case status with obese men having almost twice the risk for case status than normal weight individuals. Thus, there appears to be differences between mental distress defined as a continuous variable and mental distress defined as case status. The linear association is to some extent independent of socio-demographic factors while this is not the case for the logistic analyses of case status. It suggests that the independent association of BMI category with mental distress primarily reflects trends towards higher scores within the non-clinical range. However, alternatively the differences between the results of the linear and logistic analyses may reflect differences in statistical power.

To date, the largest study that have investigated the relation between BMI and self-reported psychiatric disorders included 177.047 individuals and reported that the prevalence of current depression, lifetime depression and anxiety varied across BMI categories and sex [3]. The highest prevalence of all three psychiatric disorders was found among women, participants with a shorter education, unemployed, and 50–59 years of age. In the present study, most associations between BMI category and mental distress case-status became non-significant when adjusting for socio-demographic factors. In contrast, Zhao et al. [3] reported that the association between BMI and self-reported psychiatric disorders existed independently of socio-demographic factors, disease status, and other lifestyle factors. The contrasting findings may be due to methodological issues including a large difference in sample size.

Interestingly, in the present study associations between BMI category and anxiety were clearly distinct from associations between BMI category and the other subscales for both men and women. More specifically, while the highest prevalence of anxiety case status was found among underweight men and obese women, severely obese women had the lowest prevalence of anxiety case status and no severely obese men reported anxiety symptoms of clinical relevance. Also, BMI category was not significantly associated with the anxiety score among women or with anxiety case status among men or women. These findings may to some extent reflect the relatively small severely obese groups, but another possible explanation is that the anxiety subscale of the SCL-90 assesses a distinct set of symptoms whereas for instance the depression subscale assesses a broader range of symptoms that may reflect poor mental health in general. Furthermore, in contrast to the well-known positive obesity-depression association, research has only found moderate evidence for a weak association between obesity and anxiety [38]. In a recent systematic review of the association between obesity and anxiety, Gariepy et al. [38] reported a pooled odds ratio of 1.4 across 14 cross-sectional studies, and it has been suggested that this odds ratio may increase with increasing obesity severity [3, 39]. However, while the majority of studies have found slightly elevated levels of anxiety in obese individuals [5, 40, 41], several have reported no association in either men or women [1, 3, 42]. Also, not all studies account for the role of anxiety subtypes. Thus, the low prevalence of anxiety observed in severely obese men and women in the present study is in contrast to prior research. The different findings may to some extent reflect study differences with respect to statistical power and adjustment for potentially confounding variables, but the exact relation between obesity and anxiety remains unclear and needs further attention.

### Effect of socio-demographic factors

Previous findings suggest that socio-demographic factors may moderate the relation between BMI and mental disorders [5] with a stronger association observed in women and individuals with long education. The stronger associations between BMI category and mental symptoms observed in men compared with women in the present study are contrasting prior research. Preliminary analyses showed that sex moderated the association between BMI group and mental distress on the somatization scale only. The stronger associations of BMI group with mental distress found in men may therefore reflect that physical complaints occurring in late midlife cause more distress among men than among women. In the present study, age did not moderate associations of BMI group and the SCL scales, but the age range of the present sample is limited and it is likely that the stronger associations between BMI group and mental distress among men than among women are specific to the late midlife population.

While none of the included socio-demographic covariates consistently moderated the relation between BMI category and mental distress, a few significant interaction effects were observed. Occupational social class influenced associations of BMI category and scores on three of four subscales for both men and women with stronger associations among men in social class 1 and on transfer income and women in the lower social classes. In a meta-analysis of thirteen general population studies Scott et al. [5] reported that both depression and anxiety were associated with obesity in women but not in men, and especially with severe levels of obesity. Interestingly, educational level influenced these associations such that the relation between obesity and depression was only observed in the subgroup with high education whereas the association between obesity and anxiety was significant in the subgroup with a low education only. Similar results were observed by Simon et al. [1], but in their study sex was not a significant moderator of the obesity-mental disorder relation and both depression and anxiety were stronger associated with obesity in the high education groups (>12 years of education). Thus, prior research is partly contrasting results from the present study and it may be speculated that obesity-related stigma is greater in men with higher income or education [5, 16]. However, considering the number of tests conducted in the present study and that the *p*-values of the moderation effects were close to the significance level (*p* = 0.05), results should be interpreted carefully. Also, in general findings are mixed and the effect of socio-demographic factors on associations of obesity and mental distress should be investigated further.

Associations between BMI category and mental distress case status were mainly explained by socio-demographic factors, especially occupational social class. Yet, differences in the prevalence of cases on the somatization subscale remained across BMI categories in men. Symptoms of somatization include the experience and communication of distress related to perceptions of bodily dysfunction for which there are no physical explanation [43]. Few previous studies have addressed the relation between obesity and somatization disorders in large community samples and results are mixed with some reporting strong associations between somatization and obesity [40] and others failing to find increased rates of somatoform disorders among obese individuals [6]. However, obesity is, in addition to a wide range of medical comorbidities [44], also associated with reduced physical function, general physical discomfort, and poor body image [45–48] that may be more impairing for men than women Thus, elevated levels of somatization observed among obese men in the current study are not surprising. Further research is highly needed.

### Limitations

Although this study has several strengths including a large population-based sample, objectively measured weight and height, and detailed socio-demographic information, some limitations should be mentioned.

By design there is very little age variation within the CAMB sample. Age has previously been found to be one of the most consistent predictors of overweight and obesity with middle-aged and older individuals in higher risk of being obese than their younger counterparts [49, 50]. Also, there is growing evidence of a negative association between age and mental distress [3, 23, 51, 52]. Members of the CAMB sample are all 49–63 years of age and thus results of the present study are restricted to midlife individuals. Furthermore, due to cultural differences that may affect public and/or individual perspectives on overweight and obesity [53], it is uncertain whether the current results can be generalized beyond the Danish population. Yet, obesity has been associated with elevated mental distress across countries [5] and findings may apply elsewhere. Also, the number of individuals in the underweight and severely obese BMI category was fairly small with 55 and 44 individuals, respectively. While it is unlikely to have had consequences for the linear analyses, the small sample sizes in these groups may have affected results from the logistic analyses.

Possible biological explanations for the observed associations between obesity and mental distress were not investigated in the present study. For instance, research has consistently found associations between elevated cytokine levels related to obesity and reduced psychological well-being [54]. Thus, such biological factors may partly explain the elevated mental distress among obese and severe obese individuals in the present study. Future studies examining the relation between BMI and mental distress should include potential underlying biological mechanisms.

The relatively modest response rate of 30% in the CAMB study should be considered when interpreting the results. For instance, it is likely that participants and non-participants differ on certain individual factors [19] with participants being more resourceful, which may explain the slightly lower SCL-90 scores in the CAMB sample when compared with Danish norms.

Finally, due to the cross-sectional design of the study, the direction of the relationship between obesity and mental distress could not be inferred from the current data. However, in general research suggests that the relation can be bidirectional in nature [12, 55]. Extensive evidence has suggested that obesity is prospectively associated with mental distress, while the opposite finding - that mental distress may cause obesity - are less consistent [56].

## Conclusion

In conclusion, we have established population-based Danish reference values for the four SCL-90 subscales somatization, depression, anxiety, and the GSI for each of the five BMI groups. Overall, results from this large late midlife community sample indicate more mental distress among underweight, obese and severely obese individuals after adjusting for age, occupational social class, and duration of education with stronger associations in men compared with women. Obese men have significantly higher risk of reporting clinical relevant symptoms of somatization compared with their normal weight counterparts independently of socio-demographic factors. Whether this association is related to a general bodily discomfort or medical conditions was not investigated and should be addressed in future research.

## Abbreviations

ANCOVA: Analyses of covariance
ANOVA: Analyses of variance
BMI: Bodymass index
CI: Confidence interval
GSI: General Severity index
M: Mean
OR: Odds ratio
SCL-90: Symptom checklist-90
SD: Standard deviation
